# Improvement of Asphalt-Aggregate Adhesion Using Plant Ash Byproduct

**DOI:** 10.3390/ma12040605

**Published:** 2019-02-18

**Authors:** Zhuangzhuang Liu, Xiaonan Huang, Aimin Sha, Hao Wang, Jiaqi Chen, Cheng Li

**Affiliations:** 1 Key Laboratory of Special Area Highway Engineering, MOE, Xi’an 710064, China; zzliu@chd.edu.cn (Z.L.); xnh0202@163.com (X.H.); cli@chd.edu.cn (C.L.); 2 School of Highway, Chang’an University, Xi’an 710064, China; 3 Rutgers, The State University of New Jersey, Piscataway, NJ 08854, USA; chenjiaqi@csu.edu.cn; 4 School of Civil Engineering, Central South University, Changsha 410083, China

**Keywords:** asphalt-aggregate adhesion, plant ash lixivium, stripping test, contact angle, interfacial transition zone

## Abstract

The adhesion bonding between asphalt and aggregate significantly influences field performance and durability of asphalt pavement. Adhesion promoters are typically used to improve asphalt-aggregate bonding and minimize moisture-related pavement damage, such as cracking and raveling. This study evaluated the effectiveness of plant ash byproduct as adhesion promoter to improve asphalt-aggregate adhesion performance. Three commonly used aggregate types (granite, basic rock, and limestone) and two asphalt binder types were used in laboratory testing. A modified stripping test method was developed to evaluate test results with image analysis and measurement of asphalt film thickness. The contact angle test and scanning electron microscopy (SEM) with energy disperse spectroscopy (EDS) were conducted. Test results showed that plant ash lixivium significantly improved asphalt-aggregate adhesion. Among three aggregate types, granite yielded the worst asphalt-aggregate adhesion for both control and treated specimens. The effectiveness of adhesion promotion varied depending on the type of asphalt or aggregate and temperature. The SEM/EDS observations showed that the mesh-like crystalline was formed at the interface between asphalt binder and aggregate in the treated specimen, which was believed to enhance the interfacial bonding and prevent asphalt film peeling off from aggregate.

## 1. Introduction

The adhesion strength of asphalt binders is an important parameter for durability of asphalt pavements. Poor bonding effect between asphalt binder and aggregate has long been identified as a major cause leading to accelerated damage of asphalt pavement [1]. When asphalt film starts peeling off from aggregates, the asphalt-aggregate adhesion strength is greatly reduced and results in debonding at the asphalt-aggregate interface [2,3]. Many previous studies have found that the damages of asphalt pavements including cracking, raveling, and permanent deformation are related to the degradation of asphalt-aggregate adhesion strength [4,5,6,7]. Therefore, to increase asphalt-aggregate adhesion strength and eventually improve the performance and durability of asphalt pavement systems, better understanding of the bonding mechanism and microstructure of asphalt-aggregate interface is needed [8,9]. 

The adhesion strength of asphalt-aggregate interface consists of three components: (1) physical bonding from mechanical interlocking, (2) physicochemical adhesion caused by surface free energy, and (3) bonding due to interfacial chemical reactions [10]. To improve asphalt-aggregate bonding, various adhesion promoters (also called anti-peeling agents) have been evaluated, such as fatty amines, organic amines, and nano admixtures [6,11,12,13]. Previous studies have found that polymer materials such as Styrene-Butadiene-Styrene (SBS), Ethylene-Vinyl-Acetate (EVA), and crumb rubber can improve moisture susceptibility of asphalt mixtures [14,15]. The promotors enhance the adhesion strength by adjusting surface energy of aggregate or forming chemical bonds between asphalt and aggregate [16]. However, most interface bonding promoters are slaked lime-based products, which are strong alkaline materials and may have adverse effects on the performance of asphalt mixtures. The previous studies mainly focused on application and evaluation of asphalt antistripping promoters (SBS, EVA, etc.) [17]. However, relatively few studies focused on evaluation of the effectiveness aggregate-promoters.

Plant ashes are byproducts from burning wheat straw, rice straw, wood straw, and corn cobs. Recent literatures reported the utilization of plant ashes (e.g., rich husk ash) in asphalt [18,19], so that the plant ash modified asphalt-based materials achieved good engineering performance. If the plant ashes were immersed in water, a large amount of alkaline substances (i.e., KOH, Ca(OH)_2_) could be released, which has the potential to enhance the asphalt-aggregate adhesion. The main chemical compositions of plant ashes are SiO_2_, K_2_O, CaO, SO_3_, MgO, Na_2_O, Fe_3_O_4_, Al_2_O_3_, and TiO_2_ [20]. However, the exact chemical compositions of plant ash lixivium vary depending on different raw materials (i.e., wheat straw, rice straw, wood straw, and corn cobs) [21,22,23,24]. The alkaline lixivium has great potential to modify the interface between asphalt and aggregate. In this study, plant ash lixivium was proposed to improve the asphalt-aggregate interface adhesion, which is rarely reported in previous literatures.

The asphalt film stripping test (also called water-boiling test) is a conventional method used to evaluate the adhesion strength of asphalt [25,26]. The test is widely used due to its simple operation procedure. The specimens after boiling are visually classified based on five-level criteria, which are not quantified criteria and could be subjective. Recently, image analysis method was concerned to provide quantitative evaluation of rolling bottle test instead of visual observation only [27]. Hence, the image analysis was combined with the traditional test in this study to investigate the effectiveness of plant ash treatment on interface bonding. In addition, advanced tests including contact angle test, SEM, and EDS were conducted to evaluate how the plant ash lixivium influence asphalt-aggregate adhesion by directly observing the microstructures of asphalt-aggregate interface zone. The observations can help explain the physicochemical adhesion mechanism of asphalt-aggregate system.

## 2. Testing Materials

### 2.1. Asphalt Binder and Aggregate

Two petroleum asphalts binders were used in this study, which were classified as #90A and #110A according to penetration test values. The basic rheological properties of two binders are provided in Table 1. The penetration, softening point, and ductility were measured based on ASTM-D5, ASTM-D36, and ASTM-D113 standards, respectively [28,29,30]. 

In this study, three types of aggregates (i.e., granite, basic rock, and limestone) were selected. For different types of aggregates, the base number and surface electrical potential related to adhesion characteristics were reported in the literature [31], as shown in Table 2.

### 2.2. Plant Ash Lixivium

**Figure 1:**
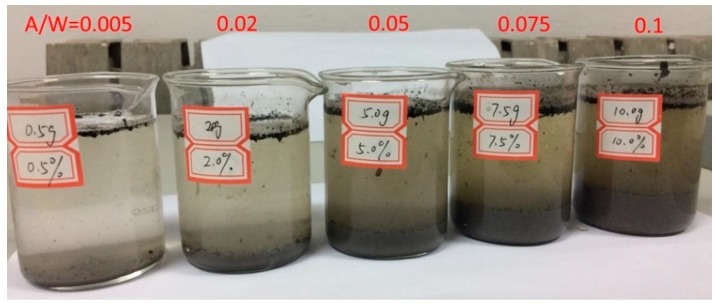
Plant ash lixivium suspensions with different ash-to-water (A/W) ratios.

The fresh plant ash used in this study was the remnant after burning rice straw, wheat straw, wood, or corn cob. The plant ash was sieved through 0.3-mm sieve and dried in oven. The dried plant ash was then mixed with deionized water (pH = 6.7 at 20 °C) and stirred for 2 mins. In the mixed suspension liquid, the plant ash released alkaline ions, which could change the pH value of the suspension. In this study, five testing specimens with different ash-to-water (A/W) mass ratios were prepared in 500-mL glass beakers, as shown in Figure 1. After 30 min defecation, the lixiviums as presented below are filtered with filter papers.

**Figure 2:**
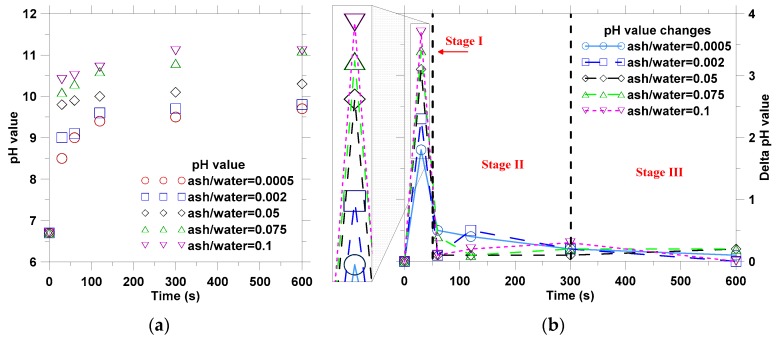
The pH value changes of suspensions with plant ash lixivium: (**a**) pH and (**b**) change of pH with time.

The pH values of suspensions were measured at 0.5, 1, 2, 5, and 10 min after stirring with the beginning of pH = 6.7 (deionized water). Figure 2 presents the changes of suspension’s pH value from stirring. The result indicated that the higher ash/water ratio yielded the higher final pH values as shown in Figure 2a. The changing trends of pH values for different suspensions were found to be similar, which showed rapid increase in the first 150 s and then remained with the relatively constant values. However, the pH value of the suspensions with high ash-water ratio increased at a faster rate than those with low ash-water ratios, and yielded higher final pH values.

In solutions, the H^+^ and OH^−^ ions concentration can be calculated based on the pH value because pH = −lg(H^+^) = 14 − lg(OH^−^). It seems the ions leaching from the plant ash could be calculated based on the pH data in Figure 2. However, in this study the solution contains K_2_CO_3_, KHCO_3_, KOH, KCl, and K_2_SO_4_ et al. It is hard to calculate the content of average ions released during the leaching, because KCl and K_2_SO_4_ rarely contributes to the pH value. However, the pH increasing must be linked to ion release, be they positive ions (K^+^, H^+^) or negative ions (OH^−^, Cl^−^, CO_3_^2−^, HCO_3_^−^, SO_4_^2−^) in this study. Hence, the increased pH values were divided by time, as shown in Figure 2b. Thereby, the higher pH value increased means the higher ions releasing rate. The results declared that the leaching process of ions from plant ash could be divided into three stages: (1) Stage I: 0–60 s when a large amount of ions are released depending on the ash/water ratio; (2) Stage II: 60–300 s when the ion releasing rate is significantly slowed; and (3) Stage III: >300 s when fewer ions could be released in plant ash lixivium.

**Figure 3:**
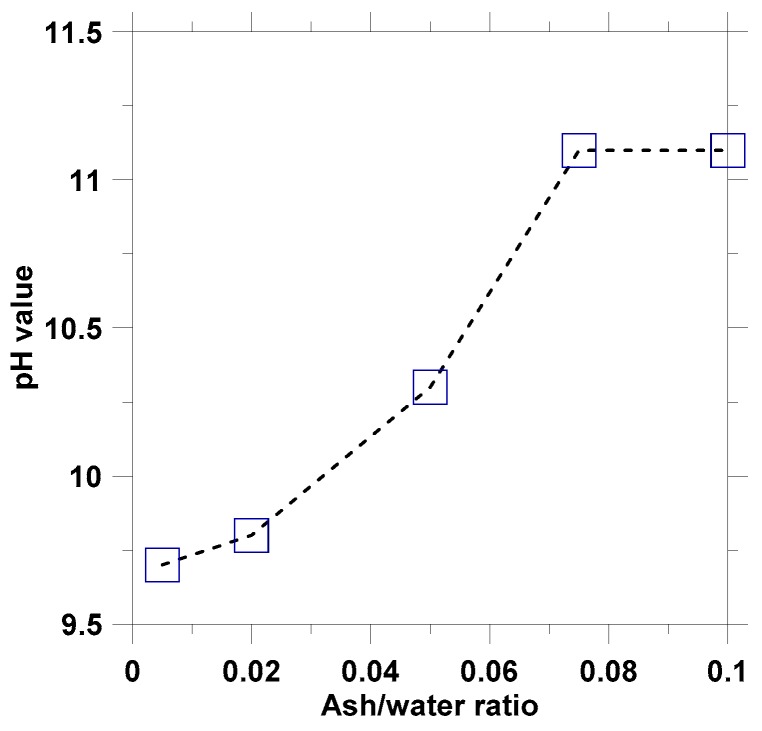
The relationship between final pH values and A/W ratios.

The relationship between the final pH values and the ash-water ratios of the suspensions was found to be nonlinear, as shown in Figure 3. The final pH value of the suspension greatly increased as the ash-water ratio increased from 0.02 to 0.075. Therefore, in this study, the plant ash lixivium was prepared using the ash/water ratio of 0.075, and the solution was defecated for 30 mins and then filtered using filter papers with 120 μm opening.

## 3. Adhesion Evaluation Using Conventional and Modified Stripping Tests

### 3.1. Conventional Stripping Test

The conventional stripping test (ASTM D3625) used to evaluate asphalt-aggregate adhesion was conducted on aggregates with particle sizes ranging from 13.2 mm to 19 mm. In this study, the stripping test was conducted on three types of aggregates (granite, limestone, and basic rock). The specimens were soaked in the plant ash lixivium for one hour and then dried in oven at 80 °C for two hours. The control specimens were prepared following the same procedure except that the aggregates were not soaked in the plant ash lixivium solution. To reduce the effect of asphalt film thickness, all the specimens were coated with asphalt binder by immersing aggregates in liquid asphalt for 45 seconds, and then hanged at room temperature for 15 mins to remove extra free binder. Then the specimens were boiled in water for 6 mins. 

After boiling, the test specimens were visually classified based on the following criteria:

Level 1: The asphalt film is completely moved by water. The removed asphalt binder is floating on water surface, and the aggregate is barely covered.

Level 2: Most of asphalt film is moved by water. The area of exposed aggregate is more than 30% of the total surface area of the specimen.

Level 3: The asphalt film can be moved by water, but most of the asphalt binder is preserved on the surface of the aggregate. The area of exposed aggregate is less than 30% of the total surface area of the specimen.

Level 4: The asphalt film is barely moved by water, but the thickness of asphalt binder is uneven. The area of exposed aggregate is less than 10% of the total surface area of the specimen.

Level 5: The asphalt film is intact, and the area of exposed aggregate is close to 0% of the total surface area of the specimen.

**Figure 4:**
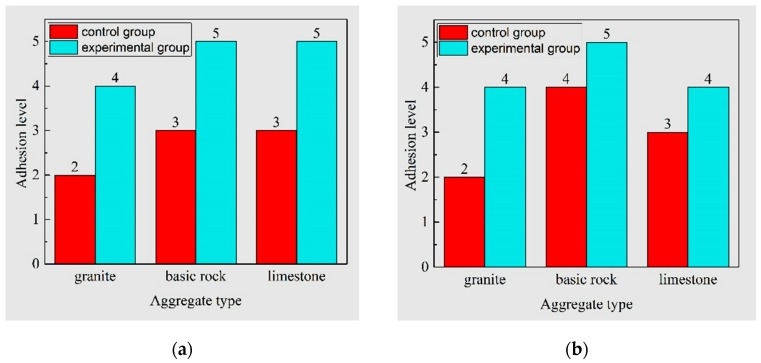
Asphalt-aggregate adhesion level based on stripping test: (**a**) #90 asphalt and (**b**) #110 asphalt (samples without treated were marked as control group, while the ones soaked with plant ash lixivium were marked as experimental group).

**Figure 5:**
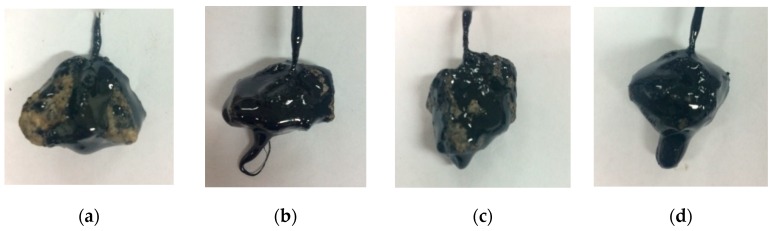
Visual observation of granite aggregates after stripping test: (**a**) #90 asphalt (control); (**b**) #90 asphalt (treated); (**c**) #110 asphalt (control); and (**d**) #110 asphalt (treated).

The conventional test results showed that the lixivium treatment can increased the adhesion bonding of different types of aggregates by increasing the rating at least one level, as shown in Figure 4. For the #90 asphalt, the adhesion performance of asphalt-limestone and asphalt-basic rock were similar, but the asphalt-granite interface showed less adhesion bonding. For the #110 asphalt, basic rock showed the best anti-stripping performance as compared to granite and limestone. In general, basic rock and limestone have better anti-stripping performance as compared to granite. It is inconclusive on the effect of asphalt type on adhesion performance due to the unknown information on chemical components of asphalt and the complex nature of asphalt-aggregate adhesion. Regardless of the asphalt or aggregate type, the effectiveness of lixivium treatment on anti-stripping was found significant. The improvement in asphalt-aggregate adhesion can also be visually observed with the appearance of aggregate after the stripping test, as shown in Figure 5.

It is noted that the conventional stripping test results determined using the five-level criteria are influenced by subjective ranking and test procedure. The conventional stripping test does not provide further comparisons between the asphalt-aggregate interfaces having the same peeling-off grade. 

### 3.2. Modified Stripping Test with Image Analysis

#### 3.2.1. Adhesion Ratio and Asphalt Film Thickness

The conventional stripping test was modified by using image analysis to quantitatively analyze test results. The conventional stripping test requires that the aggregate size should be between 13.2 mm and 19 mm. In the modified stripping test, the asphalt binder was coated on the surface of flat rectangular specimens that were cut from larger aggregates. The surface areas of rectangular specimens were found to be close to those of the spheres with approximately 18.5-mm diameter. The ranges of surface areas and the calculated equivalent sphere diameters of aggregates are shown in Table 3. 

To evaluate the specimens, two parameters were defined responding to two situations. For the specimens with aggregate surface exposure after boiling process, the adhesion ratio was defined as the ratio of the non-exposed area in the specimen surface to its total surface area, which could be determined using image analysis. For the specimens without aggregate surface exposure after boiling process, the thickness of asphalt film was used to evaluate the adhesion performance. 

**Figure 6:**
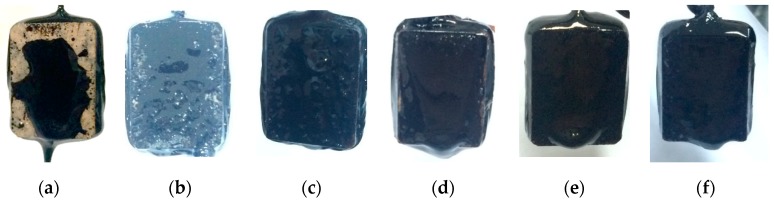
The specimens after modified stripping test: (**a**–**c**) control specimens with granite, basic rock, and limestone and (**d**–**f**) treated specimens with granite, basic rock, and limestone.

**Figure 7:**
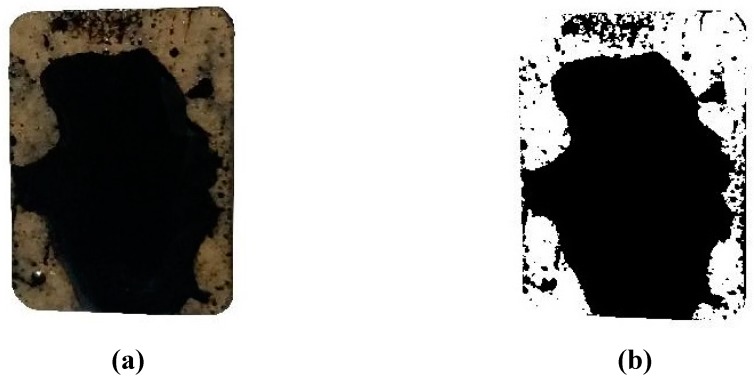
The image processing of test specimens with #110 asphalt and granite aggregate: (**a**) original image and (**b**) binary image.

Figure 6 shows the appearances of testing specimens after the stripping test. It was found that more asphalt film was peeled off from the control specimens as compared to the treated specimens. To quantify the adhesion performance, photos were taken on the front and back surfaces after the stripping test. A series of image processing techniques, including contrast enhancement and thresholding, were performed to convert the original photos to binary images, as shown in Figure 7. 

Based on the binary images, the surface areas of asphalt binder (black) and the exposed area of aggregate specimen (white) can be determined. Therefore, the adhesion ratio was calculated using Equations (1) and (2).

(1) $$\eta_{s}=\frac{\sum_{i-1}^{n}\eta_{i}}{n}$$

(2) $$\eta_{i}=\frac{X_{b}}{X_{w}+X_{b}}$$

where η_*s*_ is the adhesion ratio (opposite to peeling off ratio); η_*i*_ is the adhesion ratio of surface *i* of the specimen; *n* is the total number of surfaces of the specimen (*n* = 2 for the specimens); and *X_w_* and *X_b_* are the total number of white and black pixels in the binary image, respectively. 

**Figure 8:**
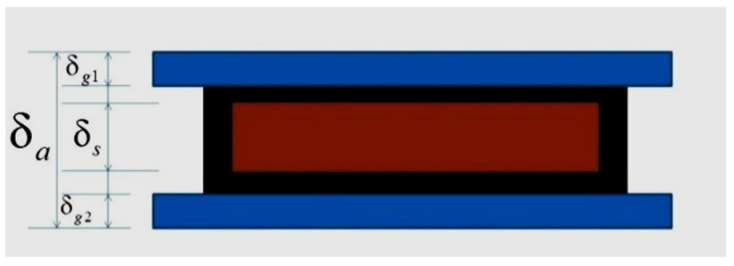
Schematic of asphalt film thickness measurement.

For the specimen without asphalt film peeling off, the average thickness of coated aggregate was measured by the caliper and glass slides, as show in Figure 8. The glass slides were slightly compressed until the asphalt film contacted with the glass slides completely. The asphalt film thickness (δ) on specimen could be calculated using Equation (3).

(3) $$\delta =\frac{\delta_{a}-\delta_{s}-(\delta_{g1}+\delta_{g2})}{2}$$

where δ_*a*_ is the total thickness of specimen with glass slides (mm); δ_*s*_ is the thickness of the specimen without asphalt film (mm); and δ_*g*1_ and δ_*g*2_ are the thickness of the glass slides (mm). 

#### 3.2.2. Adhesion Performance Results

**Figure 9:**
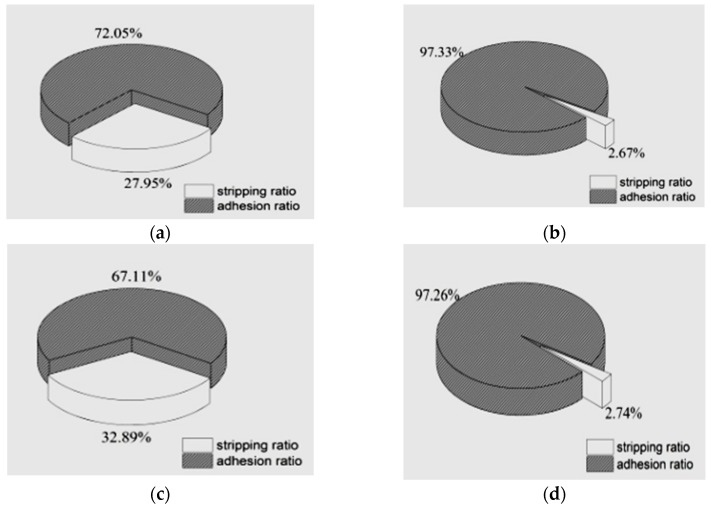
The adhesion and stripping ratios of granite specimen after modified stripping test: (**a**) control specimen with #90 asphalt; (**b**) treated specimen with #90 asphalt; (**c**) control specimen with #100 asphalt; and (**d**) treated specimen with #110 asphalt.

The test results show that the specimens with granite showed exposed aggregate surface after boiling process, while the specimens with basic rock and limestone were still fully covered by asphalt film. The image analysis results of the granite aggregate specimen with different asphalt binders are presented in Figure 9. The adhesion and stripping ratios clearly showed that the treatment using plant ash lixivium improved adhesion performance significantly for both #90 and #110 asphalt binder. For instance, the stripping ratio of granite-asphalt (#90) was reduced from 27.95% to 2.67%. At the same time, the stripping ratio of granite-asphalt (#110) changed from 32.89% to 2.74%. 

For specimens with basic rock and limestone, the conventional stripping test cannot compare the effects of treatment for basic rock and limestone specimens, because they both ranked at level 5. Hence, the modified stripping was developed providing quantitative index for adhesion performance evaluation in this case. In the modified stripping test, the thicknesses of asphalt film before and after the boiling process could be calculated. Table 4 presents the asphalt film thickness and the relative improvement for basic rock and limestone treated with plant ash lixivium, respectively. In general, the treatment of plant ash lixivium increased asphalt film thickness on aggregate surface after boiling. The treatment showed relatively higher effectiveness for the #110 asphalt in terms of the increasing ratio of asphalt film thickness. However, no consistent trend was observed for comparing the effectiveness of treatment on adhesion performance between basic rock and limestone. This is probably due to the complex mineral compositions of aggregates.

## 4. Contact Angle Test

The contact angle test is typically used to determine the balance state of three phases (solid-gas, liquid-gas, and solid-liquid) based on surface free energy theory [29,30,31,32,33,34]. The asphalt-aggregate adhesion effect was evaluated using contact angle test in this study. 

The interfacial intersection of solid-liquid surface and gas-liquid surface was defined as contact angle (*θ*), as shown in Young’s function defined in Equation (4). As shown in Equations (5)–(7), the work of adhesion (defined as *W_a_*), work of infiltration (defined as *W_i_*), and spreading coefficient (defined as *S*) increased with the reduction of contact angle (defined as *θ*), respectively. Hence, the contact angle can be used to indicate the adhesion performance between asphalt and aggregate.

(4) $$\gamma_{s\text{-}g}-\gamma_{s\text{-}l}=\gamma_{l\text{-}g}\cos\theta$$

(5) $$W_{a}=\gamma_{s\text{-}g}+\gamma_{l\text{-}g}-\gamma_{s\text{-}l}=\gamma_{l\text{-}g}(\cos\theta +1)$$

(6) $$W_{i}=\gamma_{s\text{-}g}-\gamma_{s\text{-}l}=\gamma_{l\text{-}g}\cos\theta$$

(7) $$S=\gamma_{s\text{-}g}-\gamma_{s\text{-}l}-\gamma_{l\text{-}g}=\gamma_{l\text{-}g}(\cos\theta -1)$$

where, *γ_s-g_* is the interfacial tension of solid-gas interface; *γ_s-l_* is the interfacial tension of solid-liquid interface; *γ_l-g_* is the interfacial tension of liquid-gas interface, which is unknown but can be assumed as a unit because of the invariant air and liquid phases in this study; and *θ* is the contact angle.

**Figure 10:**
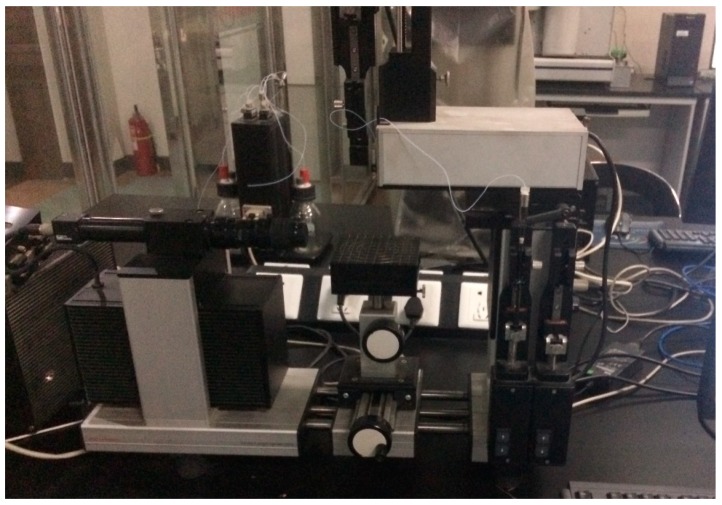
Optical video contact angle tester (OCA20).

In this study, the contact angle of asphalt-aggregate surfaces was measured using the device of OCA20 (produced by Dataphysics Group, Germany), as shown in Figure 10. The #90 asphalt was dropped onto the flat surface of rectangular-shape aggregate prepared using the same procedure described above, including washing and cutting to minimize the influences of aggregate surface roughness. The specimens were cured under 25 °C and 100 °C for 6 min, and then set under room temperature for 24 h before testing. In this study, a total of 12 aggregate specimens with 36 asphalt drops were tested.

The contact angle of each specimen was determined based on the images collected by the contact angle tester (OCA20, Dataphysics, Germany), as shown in Table 5. The results show that the contact angles of different specimens ranged between 90° and 180°, which indicates the weak adhesion at asphalt-aggregate interface in general. The smaller contact angle indicates the stronger adhesion at asphalt-aggregate interface. The specimens treated with plant ash lixivium had smaller contact angle than the control specimens, especially at 100 °C. 

**Figure 11:**
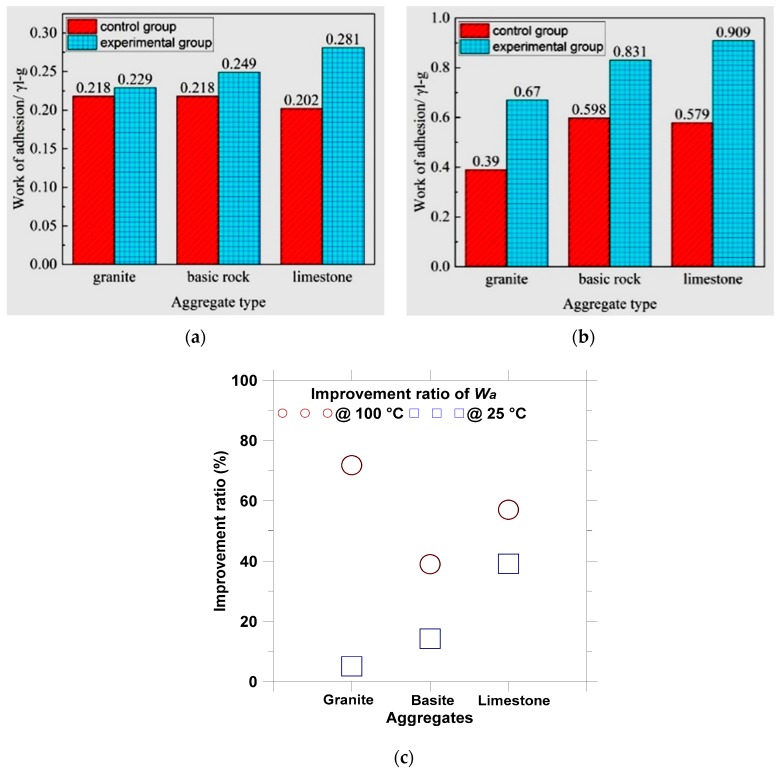
Adhesion work and improvement ratio by plant ash lixivium treatment for aggregates with #90 asphalt: (**a**) *W_a_* at 25 °C; (**b**) *W_a_* at 100 °C; and (**c**) improvement ratio.

For the aggregate-asphalt interface, the greater adhesion work (*W_a_*) needed for separating asphalt from aggregate indicates the stronger interfacial bonding between asphalt and aggregate. The calculated results of adhesion work for three aggregates with the #90 asphalt are presented in Figure 11. It is noted that the effect of plant ash lixivium should be based on the comparison of work of adhesion at the same temperature. The comparison of work of adhesion at different temperatures is not meaningful since the values of *γ_l-g_*, *γ_s-l_*, and *γ_s-g_* in Equations (5)–(7) are dependent on temperature. Therefore, the improvement ratio after the treatment of plant ash lixivium were calculated and used to evaluate the effect of temperature on treatment effectiveness.

The test results showed that the plant ash lixivium could increase adhesion work of asphalt-aggregate interface under both temperatures. For example, the adhesion work between limestone and asphalt increased from 0.202*γ_l-g_* to 0.281*γ_l-g_* at 25 °C, and from 0.579*γ_l-g_* to 0.909*γ_l-g_* at 100 °C. This means that the moisture invading process at the asphalt-aggregate interface could be delayed or prevented due to the stronger interface adhesion after treated by plant ash lixivium. 

As compared to the control specimens, the improvement ratios of adhesion work for the treated specimens with granite, limestone, and basic rock were plotted in Figure 11c. It can be concluded from the improvement ratio that high temperature, i.e., 100 °C, can promote the modification of plant ash lixivium treatment. The reason could be that asphalt become softer at relative high temperatures, and thus can flow into minor cracks or surface cavities at aggregate surface and fully coat the aggregate. In the meanwhile, the chemical reaction between asphalt and aggregate might become stronger at higher temperatures; but more evidence is needed to support this point.

## 5. Microstructure Analysis with SEM and EDS 

**Figure 12:**
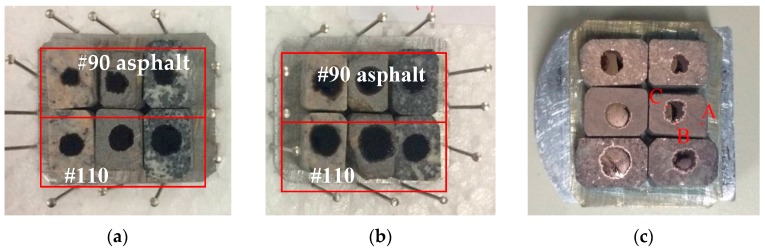
Specimens for scanning electron microscopy (SEM) with energy disperse spectroscopy (EDS) measurements: (**a**) control; (**b**) treated specimen; and (**c**) observation locations at one specimen (A, B, C).

SEM was used to observe the microstructure and element composition at the asphalt-aggregate interface using the SEM device (JSM-6390A produced by JEOL, Japan). The flat rectangular specimens of the modified stripping test specimens were cut into smaller sized specimen for SEM analysis. The hot asphalt (0.3–0.5 μL) was dropped on the control and treated aggregates, and then dried at 135 °C for 20 mins and cooled down to room temperature (25 °C) to reach steady shape. Before SEM and EDS measurements, the upper surfaces of specimens were coated with a thin layer of platinum film (5nm). The specimens with coated surfaces prepared for SEM/EDS analysis are shown in Figure 12.

The boundaries between asphalt droplet and aggregate can be clearly identified, which was the light ring around the black asphalt droplet as shown in Figure 12c. For SEM/EDS analysis, the zone close to the asphalt-aggregate boundary was divided into three areas: Area A is the uncovered aggregate; Area B is the boundary between aggregate and asphalt; and Area C is the boundary between the light ring and the black asphalt droplet. 

**Figure 13:**
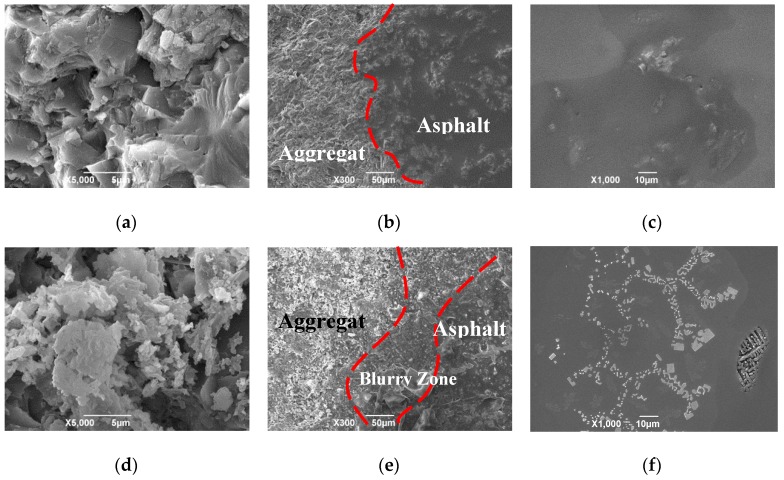
SEM images on aggregate surface, aggregate-asphalt interface, and asphalt surface of (**a**–**c**) control samples and (**d**–**f**) treated samples.

The comparison of aggregate surface images between the control and treated specimens are shown in Figure 13a,d, respectively, for A/B/C areas. It can be observed that the surface of treated aggregate has higher micro-texture that can increase surface area and thus enhance adhesion strength of asphalt-aggregate. Figure 13b and e show the clear boundary of interfacial transition zone (ITZ) between aggregate and asphalt after treatment. The thickness of ITZ was found in the range of 5–20 μm for asphalt mixture [35]. However, the ITZ of treated specimens was extended with the blurry zone, as shown in Figure 13e. The SEM images of asphalt binder surface in control specimens and treated specimens are shown in Figure 13c,f. A large amount of mesh crystallized substances were found in the treated specimens, which could increase the physiochemical effect between asphalt and aggregates. Figure 13f showed that new crystalline products were observed.

**Figure 14:**
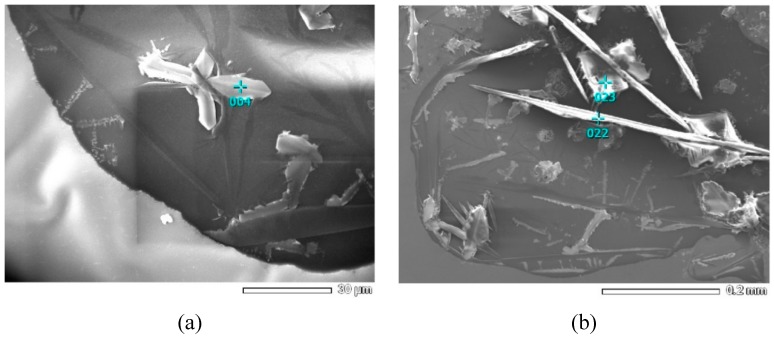
Observed crystal substances: (**a**) feather-like and (**b**) needle-like and square shape.

The SEM analysis showed that there were feather-like, needle-like, and square crystals on aggregate surfaces (Figure 14). In these images, the locations of 004, 022, and 023 at different crystals (as shown in Figure 14) were scanned by EDS, and the element composition was presented in Table 6. Although the component compositions of each burning plant ash were different, the main crystallizations of calcination were the same [22]. According to previous literature [21,22,24], the oxide compositions of the main chemical components of plant ash were SiO_2_, K_2_O and CaO, while the main crystalline compounds were generally K_2_CO_3_, KHCO_3_, K_2_SO_4_, KCl, and SiO_2_, et al. The SiO_2_ is an insoluble component and K_2_SO_4_ and KCl are neutral salts. However, K_2_CO_3_ and KHCO_3_ can be ionized following Equations (8)–(10), which can explain that the pH value of the suspensions with plant ash lixivium increased over leaching time (Figure 2).

(8) $$K_{2}{\mathrm{CO}}_{3}+H_{2}O↔2K^{+}+{\mathrm{HCO}}_{3}^{-}+{\mathrm{OH}}^{-}$$

(9) $${\mathrm{KHCO}}_{3}↔K^{+}+{\mathrm{HCO}}_{3}^{-}$$

(10) $${\mathrm{HCO}}_{3}^{-}+H_{2}O↔H_{2}{\mathrm{CO}}_{3}+{\mathrm{OH}}^{-}$$

At the observed positions (004, 022, and 023 point) in Figure 14, the atom fraction and mass fraction of elements of K, S, Cl, O, and C are shown in Table 6. It should be noted that the content of C and O could not be accepted because of the signal noise from air (including O_2_ and CO_2_). At the same time, there was no Ca element observed on specimen’s surface. It indicates that in the lixivium solution the main positive ion is K^+^. The following reasons may explain this phenomenon: (1) the lower solubility of Ca(OH)_2_ (1.65 g/L) than that of KOH (1120 g/L); (2) the CaO is sourced from CaCO_3_ which is rarely dissolved in water; and (3) there may be no calcium salts in the ashes. The results suggest that in fact the alkalization of aggregates by the plant ash lixivium treatment is mainly contributed by the ionization of K_2_CO_3_ and KHCO_3_. The alkalization promotes the higher adhesion between aggregate and asphalt binder.

**Figure 15:**
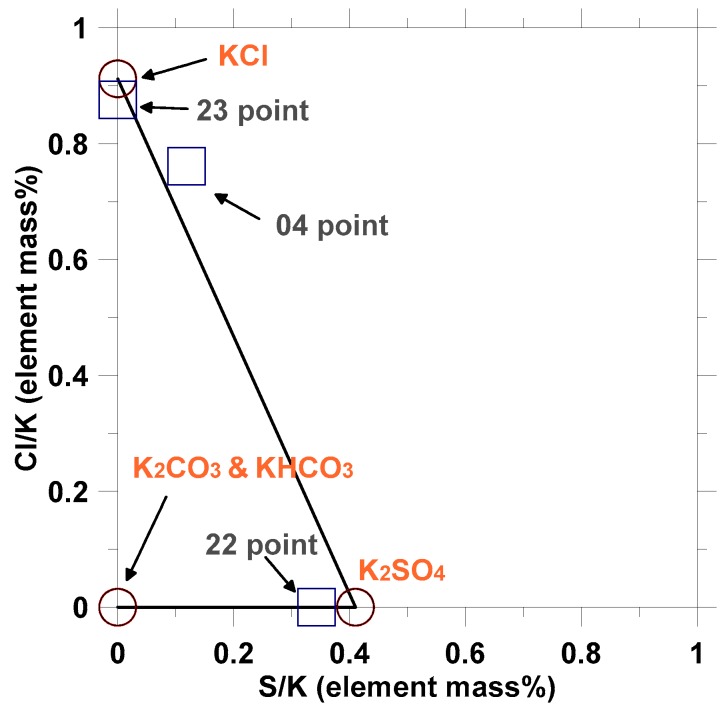
Element ratio based on analysis of EDS data.

Based on the distribution of K, S, and Cl elements, it was concluded that there was no KCl at 022 point and no K_2_SO_4_ at 023 point because S and Cl elements were not detected, respectively. Further, the element ratios of Cl/K and S/K detected by EDS were plotted in Figure 15, indicating the phase of KCl, K_2_SO_4_, and K_2_CO_3_/KHCO_3_. It was found that only KCl was present at point 023. The main phase at point 022 was K_2_SO_4_, with small content of K_2_CO_3_/KHCO_3_. At point 04, the mixture of KCl, K_2_SO_4_, and K_2_CO_3_/KHCO_3_ was found.

## 6. Conclusions

In this study, plant ash lixivium was used to improve adhesion performance of three commonly used aggregates for asphalt mixtures. To evaluate the improvement of interface adhesion, the conventional stripping test was modified with image analysis of test results. The modified stripping test results showed plant ash lixivium can effectively prevent peeling of asphalt binder from aggregate. Among three aggregate types, granite yielded the worst asphalt-aggregate adhesion for both control and treated specimens. The treatment effectiveness of plant ash lixivium varied depending on the type of asphalt and aggregate. 

The contact angle test and SEM/EDS analysis were conducted to analyze adhesion work and microstructure of aggregate-asphalt interfaces. The contact angle test revealed that plant ash lixivium increased work of adhesion of asphalt-aggregate interface, especially at high temperatures. The observations with SEM and EDS indicated that there were chemical interactions between asphalt and aggregate after the aggregate was treated with plant ash lixivium; three crystalline products were observed at the interface of asphalt and aggregate. The study findings prove the potential of using plant ashes to enhance the moisture resistance of asphalt mixtures in practical applications. 

## Figures and Tables

**Table 1:** Basic rheological properties of two asphalt binders.

Asphalt Types	Penetration at 25 °C/0.1 mm	Softening Point/°C	Ductility at 15 °C/cm
#90 grade A petroleum asphalt	88.2	45.6	>100
#110 grade A petroleum asphalt	107	44.8	>200

**Table 2:** The base number and surface potential of aggregates [31].

Aggregate Types	Granite	Limestone	Basic Rock
Base number	0.55	0.96	0.65
Surface potentials /mV	−0.31	0.62	0.04

**Table 3:** The surface area and equivalent particle size of used rectangular aggregates.

Aggregate	Calculated Surface Area/mm^2^		Equivalent Sphere Diameter/mm
	Min	Max	Average
Granite	976.37	1172.78	18.48
Basic rock	965.66	1145.95	18.32
Limestone	1032.01	1168.71	18.71

**Table 4:** The measured asphalt film thickness on aggregates and relative improvement.

Material	Average Thickness of Asphalt Film (mm)					
	#90 Asphalt			#110 Asphalt		
	Control	Treated Specimen	Improvement Ratio	Control	Treated Specimen	Improvement Ratio
Basic Rock	0.22	0.32	45.5%	0.18	0.30	66.7%
Limestone	0.20	0.30	50.0%	0.16	0.26	62.5%

**Table 5:** The test results of contact angles for different aggregates with #90 asphalt.

Aggregate Types	Control Samples		Treated Samples	
	100 °C	25 °C	100 °C	25 °C
Granite	128°	141°	109°	140°
Basic rock	114°	141°	100°	139°
Limestone	115°	143°	95°	136°

**Table 6:** The energy disperse spectroscopy (EDS) analysis results of different crystals on aggregate surface.

Location	Index	C	O	S	Cl	K
004 point	Mass/%	21.87	2.47	4.78	30.64	40.25
	Atom/%	45.32	3.84	3.71	21.51	25.62
022 point	Mass/%	5.59	26.33	17.39	--	50.69
	Atom/%	11.78	41.67	13.73	--	32.82
023 point	Mass/%	--	--	--	46.69	53.31
	Atom/%	--	--	--	49.14	50.86
